# Supplementary material for: Chromothripsis during telomere crisis is independent of NHEJ, and consistent with a replicative origin
Source: Genome Res. 2019 May;29(5):737–49. doi: 10.1101/gr.240705.118 (PMC6499312; doi:10.1101/gr.240705.118)
Supplement: Supplemental Material [file supp_gr.240705.118_Supplemental_file_1.zip › contigs/annotated_contigs/DB79/contig.2.DB79_length_376_mean_cov_15.710106383.docx]

**DB79_length_376_mean_cov_15.710106383**

```
TCTCCTCAAGATATCATTAAGAATAAGATCTTACCTGCATTTGTTTCTCAGTCCTCTGGTGTCAAGTCCTTCTCCTTGAACCATTCAGT
 >chr19:42357368-42357590 + E=3e-122
ACATAGGAAAACCTGTTGAGCTATTCCAAAGACATGAAGCCTGGAGACCCCCTTCCCATTCCCACCCTTAGAAGGGGCTCAAGGGATGT

GCTTGATCACTGATTCTTTTTCCTGATGTTACCTACACCCCTT|G|CCTGGGCAACACGGTGAAACCCCCTCTCTACTAAAATACAAAA
                                            >chr19:42360771-42360926 - E=7e-82
AATTAGGCCGGGCGCGGTGGCTCACGCCTGTAATCCCAGCACTTTGGGAGGCCGAGGCGGGCGGATCACGAGGTCAGGAGATGGAGACC

ATCCTGGCTAACATGGTGAAAC
```
